# The Impact of COVID-19 on Suicidal Behavior in King Abdulaziz Medical City in Saudi Arabia

**DOI:** 10.7759/cureus.62057

**Published:** 2024-06-10

**Authors:** Meshal R Alotaibi, Ibrahim A Alsuwailem, Khalid Alsultan, Khalid S Alwasem, Ziad S AlSaadi, Hatim Assiri, Laila Layqah, Joharah Almubrad, Noura Gammash, Reem Al-Qahtani, Meshal Alaqeel

**Affiliations:** 1 Adult Mental Health, King Abdulaziz Medical City, Riyadh, SAU; 2 Psychiatry, Prince Mutaib Bin Abdulaziz Hospital, Sakaka, SAU; 3 Child and Adolescent Psychiatry, King Abdullah Specialist Children Hospital, Riyadh, SAU; 4 Research, King Abdullah International Medical Research Center, Riyadh, SAU; 5 Psychiatry, Mental Health, Ministry of the National Guard Health Affairs, Riyadh, SAU; 6 Internal Medicine, King Khalid University, Abha, SAU; 7 Mental Health, Ministry of the National Guard Health Affairs, Riyadh, SAU

**Keywords:** covid-19, suicidal attempts, emergency medical service, suicidal behavior, psychiatry and mental health

## Abstract

Introduction: During the COVID-19 pandemic, there has been a global increase in mental health issues, including suicidal behaviors. This study focuses on assessing the impact of the pandemic on the prevalence and characteristics of suicidal behavior at King Abdulaziz Medical City in Riyadh, Saudi Arabia.

Methods: A retrospective cohort study was conducted on 580 patients from January 2017 to December 2022, focusing on those aged 18 and above referred for suicide evaluation. Data were collected via chart reviews and analyzed using Statistical Product and Service Solutions (SPSS, version 25; IBM SPSS Statistics for Windows, Armonk, NY).

Results: Our study reviewed 580 patient charts, with 555 (95.7%) meeting the inclusion criteria. The majority of participants were in the 18-29 age group (66.7%). We observed an increase in the number of cases undergoing suicide attempt assessment post COVID-19, with 296 incidents (53.3%) from March 2020 to December 2022 (about a year and eight months), compared to 259 incidents (46.7%) from January 2017 to March 2020 (over three years). A significant post-pandemic increase was noted in individuals with secondary education or higher (p = 0.004). No significant changes were found in other demographic variables or in the profiles of individuals with an intention to end life before and after the pandemic.

Conclusion: The study highlights the nuanced impact of the COVID-19 pandemic on suicidal behavior in Saudi Arabia, revealing an increased demand for suicide assessments, particularly among educated individuals. However, no corresponding increase was observed in the rate of high-intent suicidal cases or other significant variables. The findings underscore the complexity of factors influencing suicidal behavior during the pandemic and the need for targeted mental health interventions. Future research, ideally supported by a national database, is essential for a comprehensive understanding of suicidal behavior in Saudi Arabia.

## Introduction

The emergence of the COVID-19 pandemic, first identified in China towards the end of 2019, has precipitated a global health crisis of unprecedented scale [1]. Recognizing the severe implications of the virus's rapid dissemination, the World Health Organization (WHO) declared it a pandemic on March 11, 2020, highlighting the alarming levels of spread and severity [2]. In response, countries worldwide have adopted various public health measures, ranging from mandatory stay-at-home orders to the temporary shutdown of educational institutions, along with enforcing travel restrictions and advocating for social and physical distancing. Numerous nations have also acknowledged the situation as a public health emergency of international concern [3].

In Saudi Arabia, the initial case of COVID-19 was reported on March 2, 2020 [4]. Subsequently, from late March until June 2020, the government imposed a lockdown in major cities, including Riyadh, and enforced travel restrictions across the kingdom [5]. Although the direct physical health impacts of the virus are evident, its ramifications on mental health globally are equally significant and distressing [6-8]. Emerging research indicates a marked decline in psychological well-being and an uptick in anxiety and depression compared to pre-pandemic levels, with these adverse mental health effects persisting beyond the pandemic's peak [9-12].

The well-established link between mental illness, depression, and suicide underscores the gravity of this situation. Globally, suicide accounts for over 800,000 deaths annually, ranking as the second leading cause of death among individuals aged 15-29. For every suicide incident, approximately 20 other individuals attempt it [13]. Studies such as those by Stack et al. have found a correlation between the extent of social isolation during pandemics, such as the 1918 Spanish flu, and increased suicide rates, regardless of the pandemic's mortality severity [14]. Similarly, Shobhana et al. observed diverse global trends in suicidal thoughts, ideations, and self-harming behaviors during the COVID-19 pandemic, with some countries reporting consistent levels, while others, such as Bangladesh and France, witnessed an escalation in suicide rates [15].

In the context of Saudi Arabia, there has been limited national research on pandemic-related suicides. However, Almaghrebi's study during the three-month COVID-19 lockdown in the Kingdom highlighted risk factors associated with 29 suicide attempts. These included psychological distress, interpersonal conflicts, domestic violence, unemployment, financial challenges, and intense fear of infection, with the lockdown disproportionately impacting women, especially domestic abuse victims and those with mental health issues [16].

Global studies have illustrated varied trends in suicide rates during the COVID-19 pandemic. In the United States, there was a notable decrease in suicide rates in 2020, particularly among non-Hispanic Whites, with a national decline of approximately (3%) and a more pronounced (4.5%) drop within this demographic. However, this trend did not extend to all groups; minority populations, including Hispanic males, non-Hispanic multiracial females, and American Indian and Alaska Native males, experienced increases in suicide rates [17-20]. In contrast, Japan experienced a significant increase in suicide rates during the pandemic's second wave, with a (16%) rise. This increase coincided with heightened unemployment rates, indicating a possible ecological correlation and reinforcing the established link between economic challenges and suicide rates [21-24].

In India, the year 2020 saw a substantial escalation in suicide rates, marked by an (18%) increase among males and a (5%) rise among females. The increase was particularly acute in males from low sociodemographic index states and in both genders from high sociodemographic index states [25]. Similarly, Nepal reported significant increases in suicide rates, most notably in its poorest provinces, which are home to many migrant workers [26]. Brazil's experience was more complex, with an overall (13%) reduction in national suicide rates, yet specific demographic groups in deprived regions saw significant increases [27]. In contrast, Ecuador did not observe an overall rise in police-reported suicides, and there was a noted decrease in such cases among indigenous people and other ethnic minority groups [28].

Given this background, the present study aims to elucidate the impact of the COVID-19 pandemic on suicidal behavior, with a specific focus on King Abdulaziz Medical City in Riyadh, Saudi Arabia. It seeks to analyze the prevalence and nature of psychiatric consultations linked to suicide before and during the pandemic, assess the associated sociodemographic factors, and examine the broader implications of these findings.

## Materials and methods

Study population and sample

This retrospective cohort study included all patients identified through psychiatric assessments at King Abdulaziz Medical City -Riyadh, who exhibited suicidal ideation or engaged in suicidal behaviors. The study encompassed a sample size of 580 individuals. The inclusion criteria were persons aged 18 years or older, referred to mental health services between January 2017 and December 2022, for suicide evaluation or with recorded suicidal attempts. Exclusion criteria included individuals under 18 and referrals not primarily for suicidal evaluation.

Data collection method

The study employed a retrospective chart review approach, utilizing electronic medical records from King Abdulaziz Medical City as the primary data source. Data were collected and verified against inclusion and exclusion criteria using a structured Case Report Form (CRF). The CRF was divided into four sections: (1) demographic characteristics (age, gender, socioeconomic status, and education level); (2) presentation details (referral reason, referring department, and history of mental disorders); (3) medication history; and (4) outcomes of the evaluation.

Statistical analysis

The data were analyzed with Statistical Package for Social Sciences (SPSS, version 25; IBM SPSS Statistics for Windows, Armonk, NY). Categorical data (e.g., gender, education level, social and employment status) were presented by frequencies and percentages. Continuous variables such as length of stay were presented by the interquartile range (IQR) and median. Descriptive and inferential statistics have been performed for the socio-demographic and psychological variables. The categorical variables were compared using a chi-square test or Fisher’s exact test or Mann-Whitney test, as appropriate. All tests were two-tailed, and significance was accepted at a p-value < 0.05.

## Results

**Figure 1:**
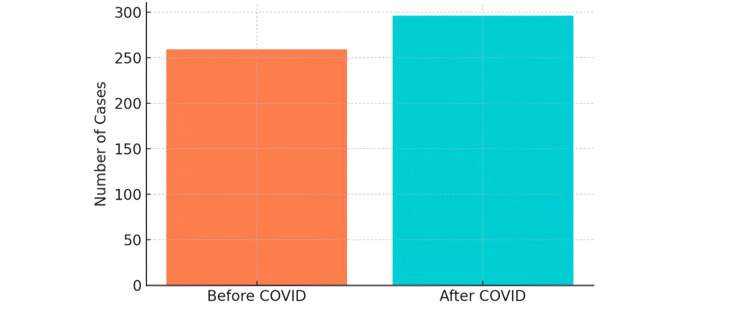
Number of cases for suicide assessment before and after COVID-19

Our study, spanning from January 2017 to December 2022, involved a review of 580 patient charts, with 555 charts (95.7%) meeting the inclusion criteria. The remaining 25 charts (4.3%) lacked critical data and were excluded from the analysis. The demographic breakdown showed a predominance of the 18-29 age group, representing 370 individuals (66.7%), indicating a younger cohort within the study population. Females constituted the majority of the study participants, with 355 cases (64%). A majority of participants were single (312, 56.2%), and the most common education level was secondary (335, 60.4%; Table 1). Post-COVID-19, from March 23, 2020, to December 2022, we observed an increase in cases with 296 (53.3%) compared to 259 (46.7%) before the pandemic, from January 2017 to March 23, 2020 (Figure 1).

**Table 1:** Sociodemographic characteristics of the study participants

Variable	Frequency	Percentage
	N=555	
Age		
18-29	370	66.7
30-39	93	16.8
40-49	47	8.5
50-59	23	4.1
> 60	22	4
Gender		
Male	200	36
Female	355	64
Level of education		
Primary	54	9.7
Intermediate	54	9.7
secondary	335	60.4
Higher education	98	17.6
Not documented	14	2.5
Marital status		
Single	312	56.2
Married	182	32.8
Divorced/widow	61	11
Have you ever been diagnosed with a psychiatric illness?		
Never	158	28.5
Yes	379	71.5
Employment status		
Unemployed	131	23.6
Employed	424	76.4
1. Cases undergoing suicidal attempt assessment before COVID ( January 2017 to 23 March 2020)	259	46.7
2. Cases undergoing suicidal attempt assessment after COVID ( 24 March 2020 to December 2022)	296	53.3

When analyzing the difference in the timeline in relation to the COVID-19 pandemic, there was a significant difference post pandemic in the level of education, with an increase in participants who had completed secondary education and higher (p=0.004). No significant differences were observed in other sociodemographic variables, including age, gender, marital status, employment status, or outcomes (p>0.05 for each).

For individuals with an intention to end their life, the study showed no significant changes post pandemic in demographic or clinical profiles. The age distribution, predominantly within the 18-29 range, gender ratio, marital status, and employment status, remained stable (p>0.05 for each). The outcomes of psychiatric assessments, which included admissions or discharges, were consistent with pre-pandemic patterns (p=0.984). However, before the COVID-19 pandemic, 18 out of 86 individuals (21%) from the 'intention to end life' group were admitted under psychiatry. In contrast, considering all cases referred during the same period, 26 out of 259 cases (10%) were admitted under psychiatry, indicating that a significant proportion of high-intent cases received psychiatric admissions. Post COVID, this pattern persisted with similar proportions - 13 out of 61 individuals (21.3%) in the 'intention to end life' group were admitted under psychiatry, while out of all cases referred in this period, 25 out of 296 (8.4%) received psychiatric admissions (Table 2).

**Table 2:** Number of cases referred to the psychiatry emergency for suicide assessment before and after the COVID-19 crisis

Variable	Before COVID	After COVID	P value
	N=259	N=296	
Age			
18-29	168 (64.8)	202 (68.2)	
30-39	47 (18.1)	46 (15.5)	0.293
40-49	18 (6.9)	29 (9.8)	
50-59	12 (4.6)	11 (3.7)	
> 60	14 (5.4)	8 (2.7)	
Gender			
Male	162 (62.5)	193 (65.2)	0.516
Female	97 (37.5)	103 (37.8)	
Level of Education			
Primary	30 (11.6)	24 (8)	
Intermediate	31 (12)	23 (7.8)	0.004
Secondary	150 (58)	185 (62.5)	
Higher education	36 (13.9)	62 (21)	
Not documented	12 (4.6)	2 (0.6)	
Marital status			
Single	140 (54)	172 (58)	0.22
Married	90 (34.7)	92 (31)	
Divorced/widow	29 (11.2)	32 (10.8)	
Employment status			0.33
Unemployed	66 (25.5)	65 (22)	
Employed	193 (74.5)	231 (78)	
Outcome			
	74 (28.5)	106 (35.8)	0.816
Admission under medicine	26 (10)	25 (8.4)	
	159 (61.4)	165 (55.7)	
Admission under psychiatry			
Discharge			
Intention to end life			
					Before COVID	After COVID	P value
N = 86	N = 61						
Age			
18-29	55 (64)	38 (62.3)	
30-39	16 (18.6)	13 (21.3)	0.807
40-49	4 (4.6)	5 (8.2)	
50-59	5 (5.8)	2 (3.3)	
> 60	6 (7)	3 (5)	
Gender			
Male	51 (59.3)	40 (65.6)	0.44
Female	35 (40.7)	21 (34.4)	
Level of Education			
Primary	10 (11.6)	7 (11.5)	
Intermediate	11 (12.8)	11 (18)	0.335
secondary	50 (58)	34 (55.7)	
Higher education	10 (11.6)	9 (14.7)	
Not documented	5 (5.8)	0	
Marital status			
Single	50 (58)	36 (59)	0.393
Married	27 (31.4)	18 (29.5)	
Divorced/widow	9 (10.5)	7 (11.5)	
Employment status			
Unemployed	69 (80.2)	49 (80.3)	0.98
Employed	17 (19.7)	12 (19.7)	
Outcome			
Admission under medicine	28 (32.5)	19 (31.1)	
			0.984
Admission under psychiatry	18 (21)	13 (21.3)	
Discharge	40 (46.5)	29 (47.5)	

## Discussion

Considering that our chart review identified 296 cases of suicidal attempts from March 23, 2020, to December 2022 compared to 259 cases from January 2017 to March 2020, our study observed an increase in suicide cases post COVID. This increase aligns with the global trends observed in countries such as Japan, where there was a significant surge in suicide rates [21,22], and with the historical pattern of increased suicide rates following epidemics and economic downturns (14,23,24). However, it contrasts with the trends in the United States and Brazil, where a decrease in suicide rates was noted [17-20,27]. Causes were varied in nature; for instance, in a US study, they were an alarming disproportionate elevation of suicidal attempts only among racial minorities [17]. Moreover, in Brazil, they argued the reasoning behind the decrease likely due to COVID-19's positive effects of the epidemic (greater time with family, increased social cohesion, decreased academic fees, and problems with peers in school) on suicides would be nullified, thus leading to a decrease in the cases [27].

In our study, we observed a significant increase in suicidal behavior among individuals with secondary and higher education levels after the COVID-19 pandemic. This aligns with the findings of Peng et al.'s meta-analysis, which demonstrated a heightened prevalence of mental health issues, including depression, anxiety, and suicidal ideation, among medical students worldwide during the pandemic [29]. The meta-analysis reported an exceptionally high prevalence of depression (41%), anxiety (38%), and suicidal ideation (15%) among medical students, suggesting an intensified mental burden in this demographic. Notably, the rates of these mental health issues were found to be higher than those in the general population during the same period. This correlation suggests that the pandemic may have had a more profound psychological impact on individuals engaged in higher education, potentially due to factors such as increased academic pressures, uncertainty about the future, and disruption of normal educational activities.

In a systematic review and meta-analysis assessing the impact of the COVID-19 pandemic on suicidal ideation, attempts, and deaths, which included 45 studies with 67 samples, there was a notable increase in both suicidal ideation and suicide attempts during the pandemic [30]. Specifically, the prevalence of suicidal ideation more than doubled, with a prevalence ratio (PR) of 2.014 (95% CI: 1.604-2.529), indicating an increase of about (101.4%). The prevalence of suicide attempts also increased, with a PR of 1.218 (95% CI: 1.089-1.362), representing a (21.8%) increase. These increases were observed among both non-clinical and clinical samples. Despite these significant increases in suicidal ideation and attempts, the rate of death by suicide remained largely unchanged. The study attributes these trends to the profound psychological, social, and economic impacts of the pandemic, including increased mental health issues and economic stressors. In our study, we observed a decrease in the number of individuals with an intention to end their life, from 86 before the pandemic to 61 afterwards. This suggests that, while the overall number of referred cases for suicide assessment increased post-pandemic, the proportion of serious suicidal cases may not have risen correspondingly. It also implies that, post pandemic, there may have been an increase in expressions of suicidal ideation among individuals, even if their actual intention to end their lives was not as pronounced.

In our study conducted at King Abdulaziz Medical City, Riyadh, where no dedicated psychiatric ward exists, we noted a distinctive pattern in the management of cases involving the intention to end life. Cases that are deemed low risk, necessitating brief observation, or presenting with medical complications are typically admitted under general medicine or discharged for clinic follow-up. The more severe cases, however, are transferred to specialized psychiatric hospitals to be admitted under psychiatry and to ensure patient safety. Before the pandemic, 18 out of 86 individuals (21%) from the 'intention to end life' group were admitted under psychiatry, compared to 26 out of 259 cases (10%) from all referred cases. Post COVID, these figures were 13 out of 61 (21.3%) and 25 out of 296 (8.4%), respectively. This suggests that, while the rate of psychiatric admissions remained stable for high-intent cases, the overall rate of psychiatric admissions among all referred cases decreased slightly post pandemic.

Several limitations in our study should be noted. First, the absence of a dedicated psychiatric ward in the hospital led to the transfer of severe cases to external psychiatric facilities. This lack of in-house psychiatric services might have also deterred the arrival of high-risk patients, potentially resulting in an underrepresentation of severe cases in our analysis. Second, our approach relied on a retrospective chart review, which is subject to the inherent limitations of historical data accuracy and completeness. Third, the specific sociocultural and healthcare context of Saudi Arabia, particularly in King Abdulaziz Medical City, may limit the generalizability of our findings to other regions or healthcare systems. Finally, the evolving nature of the COVID-19 pandemic and its varying impact over time introduces an element of temporal variability that might not be fully captured in this study. These factors should be considered when interpreting our findings. Future studies would benefit from the establishment of a national database, enabling a comprehensive understanding of the demographics and long-term outcomes of patients with suicidal behavior across Saudi Arabia.

## Conclusions

In conclusion, our study at King Abdulaziz Medical City provides a nuanced understanding of the impact of the COVID-19 pandemic on suicidal behavior in Saudi Arabia. We observed a modest increase in the overall referred cases for suicide assessment post pandemic. However, the rate of individuals with an intention to end life did not show a corresponding increase, suggesting a complex interplay of factors influencing suicidal behavior during the pandemic involving negative effects (fears about the pandemic, family economic difficulties, limited access to basic health facilities, and reduced social contacts).

The establishment of a national database would be a significant step forward, offering a more comprehensive view of the demographics and long-term outcomes of patients with suicidal behavior. Such an approach would enhance our understanding and ability to effectively address this critical public health issue, not only in the context of global crises such as a pandemic but also in more stable times.

## References

[1] Sher L (2020). The impact of the COVID-19 pandemic on suicide rates. QJM.

[2] Cucinotta D, Vanelli M (2020). WHO declares COVID-19 a pandemic. Acta Biomed.

[3] Jernigan DB (2020). Update: Public health response to the coronavirus disease 2019 outbreak - United States, February 24, 2020. MMWR Morb Mortal Wkly Rep.

[4] (2024). COVID-19 dashboard of Saudi Arabia. https://www.moh.gov.sa/en/Pages/default.aspx.

[5] (2024). Custodian of the Two Holy Mosques issues curfew order to limit spread of Novel Coronavirus from seven in the evening until six in the morning for 21 days starting in the evening of Monday 23 March. Saudi Press Agency.

[6] Panchal N, Kamal R, Orgera K (2020). The implications of COVID-19 for mental health and substance use. Semantic Scholar.

[7] Li S, Wang Y, Xue J, Zhao N, Zhu T (2020). The impact of COVID-19 epidemic declaration on psychological consequences: a study on active Weibo users. Int J Environ Res Public Health.

[8] Qiu J, Shen B, Zhao M, Wang Z, Xie B, Xu Y (2020). A nationwide survey of psychological distress among Chinese people in the COVID-19 epidemic: implications and policy recommendations. Gen Psychiatr.

[9] Vindegaard N, Benros ME (2020). COVID-19 pandemic and mental health consequences: systematic review of the current evidence. Brain Behav Immun.

[10] Benke C, Autenrieth LK, Asselmann E, Pané-Farré CA (2020). Lockdown, quarantine measures, and social distancing: associations with depression, anxiety and distress at the beginning of the COVID-19 pandemic among adults from Germany. Psychiatry Res.

[11] Ozamiz-Etxebarria N, Dosil-Santamaria M, Picaza-Gorrochategui M, Idoiaga-Mondragon N (2020). Stress, anxiety, and depression levels in the initial stage of the COVID-19 outbreak in a population sample in the northern Spain. Cad Saude Publica.

[12] Pierce M, Hope H, Ford T (2020). Mental health before and during the COVID-19 pandemic: a longitudinal probability sample survey of the UK population. Lancet Psychiatry.

[13] (2024). Suicide worldwide in 2019. https://www.who.int/publications/i/item/9789240026643.

[14] Stack S, Rockett IR (2021). Social distancing predicts suicide rates: analysis of the 1918 flu pandemic in 43 large cities, research note. Suicide Life Threat Behav.

[15] Shobhana SS, Raviraj KG (2022). Global trends of suicidal thought, suicidal ideation, and self-harm during COVID-19 pandemic: a systematic review. Egypt J Forensic Sci.

[16] Almaghrebi AH (2021). Risk factors for attempting suicide during the COVID-19 lockdown: identification of the high-risk groups. J Taibah Univ Med Sci.

[17] Mitchell TO, Li L (2021). State-level data on suicide mortality during COVID-19 quarantine: early evidence of a disproportionate impact on racial minorities. Psychiatry Res.

[18] Bray MJ, Daneshvari NO, Radhakrishnan I, Cubbage J, Eagle M, Southall P, Nestadt PS (2021). Racial differences in statewide suicide mortality trends in Maryland during the coronavirus disease 2019 (COVID-19) pandemic. JAMA Psychiatry.

[19] Larson PS, Bergmans RS (2022). Impact of the COVID-19 pandemic on temporal patterns of mental health and substance abuse related mortality in Michigan: an interrupted time series analysis. Lancet Reg Health Am.

[20] Ehlman DC, Yard E, Stone DM, Jones CM, Mack KA (2022). Changes in suicide rates - United States, 2019 and 2020. MMWR Morb Mortal Wkly Rep.

[21] Tanaka T, Okamoto S (2021). Increase in suicide following an initial decline during the COVID-19 pandemic in Japan. Nat Hum Behav.

[22] Horita N, Moriguchi S (2022). Trends in suicide in Japan following the 2019 coronavirus pandemic. JAMA Netw Open.

[23] Barr B, Taylor-Robinson D, Scott-Samuel A, McKee M, Stuckler D (2012). Suicides associated with the 2008-10 economic recession in England: time trend analysis. BMJ.

[24] Reeves A, Stuckler D, McKee M, Gunnell D, Chang S-S, Basu S (2012). Increase in state suicide rates in the USA during economic recession. Lancet.

[25] Arya V, Page A, Spittal MJ (2022). Suicide in India during the first year of the COVID-19 pandemic. J Affect Disord.

[26] Acharya B, Subedi K, Acharya P, Ghimire S (2022). Association between COVID-19 pandemic and the suicide rates in Nepal. PLoS One.

[27] Orellana JDY, de Souza MLP (2022). Excess suicides in Brazil: inequalities according to age groups and regions during the COVID-19 pandemic. Int J Soc Psychiatry.

[28] Gerstner RM, Narváez F, Leske S, Troya MI, Analuisa-Aguilar P, Spittal MJ, Gunnell D (2022). Police-reported suicides during the first 16 months of the COVID-19 pandemic in Ecuador: a time-series analysis of trends and risk factors until June 2021. Lancet Reg Health Am.

[29] Peng P, Hao Y, Liu Y (2023). The prevalence and risk factors of mental problems in medical students during COVID-19 pandemic: a systematic review and meta-analysis. J Affect Disord.

[30] Yan Y, Hou J, Li Q, Yu NX (2023). Suicide before and during the COVID-19 pandemic: a systematic review with meta-analysis. Int J Environ Res Public Health.

